# The hyperinflammatory spectrum: from defects in cytotoxicity to cytokine control

**DOI:** 10.3389/fimmu.2023.1163316

**Published:** 2023-04-28

**Authors:** Raquel Planas, Matthias Felber, Stefano Vavassori, Jana Pachlopnik Schmid

**Affiliations:** ^1^ Division of Immunology, University Children’s Hospital Zurich, Zurich, Switzerland; ^2^ Department of Cell Biology, Physiology and Immunology, University of Barcelona, Barcelona, Spain; ^3^ Pediatric Immunology, University of Zurich, Zurich, Switzerland

**Keywords:** hemophagocytic lymphohistiocytosis, inflammation, hyperinflammatory syndromes, cytotoxic lymphocytes, cytokines, immune dysregulation, inborn errors of immunity

## Abstract

Cytotoxic lymphocytes kill target cells through polarized release of the content of cytotoxic granules towards the target cell. The importance of this cytotoxic pathway in immune regulation is evidenced by the severe and often fatal condition, known as hemophagocytic lymphohistiocytosis (HLH) that occurs in mice and humans with inborn errors of lymphocyte cytotoxic function. The clinical and preclinical data indicate that the damage seen in severe, virally triggered HLH is due to an overwhelming immune system reaction and not the direct effects of the virus *per se*. The main HLH-disease mechanism, which links impaired cytotoxicity to excessive release of pro-inflammatory cytokines is a prolongation of the synapse time between the cytotoxic effector cell and the target cell, which prompts the former to secrete larger amounts of cytokines (including interferon gamma) that activate macrophages. We and others have identified novel genetic HLH spectrum disorders. In the present update, we position these newly reported molecular causes, including CD48-haploinsufficiency and ZNFX1-deficiency, within the pathogenic pathways that lead to HLH. These genetic defects have consequences on the cellular level on a gradient model ranging from impaired lymphocyte cytotoxicity to intrinsic activation of macrophages and virally infected cells. Altogether, it is clear that target cells and macrophages may play an independent role and are not passive bystanders in the pathogenesis of HLH. Understanding these processes which lead to immune dysregulation may pave the way to novel ideas for medical intervention in HLH and virally triggered hypercytokinemia.

## 1 Introduction

Over the last few years, the use of whole-exome and whole-genome sequencing has broadened the spectrum of HLH disorders. In-depth characterization of genetic causes and related molecular dysfunctions in hemophagocytic lymphohistiocytosis (HLH) provides us with a better understanding of immune regulation processes. HLH is a unique clinical entity. Primary forms of HLH (pHLH) are caused by genetic defects that impair lymphocytes’ cytotoxic machinery. For instance, individuals with deleterious mutations in the gene coding for perforin (*PRF1*) develop HLH (1, 2). pHLH disorders include autosomal recessive mutations in genes involved in the perforin-dependent cytotoxic lymphocyte granule release, causing familial HLH (FHL) and other genetic defects such as *RAB27A*, *LYST* and *SH2D1A* mutations. Moreover, HLH-like manifestations can occur in association with other genetic defects, i.e. inborn errors of immunity (IEI). In these cases, HLH is often considered as an acquired event secondary to the concomitant disease, even though patients may fulfil the HLH diagnostic criteria (3).

Primary forms of HLH are distinct from autoinflammatory diseases. Despite clinical manifestations can be similar, posing a challenge to the clinicians during initial assessments, the genetics and underlying causes of pHLH and autoinflammatory diseases are different. Autoinflammatory diseases are caused by dysregulation that mainly affect the innate immune system and the activation of pro-inflammatory pathways; they include inflammasomopathies, interferonopathies, and a group of non-inflammasome-related diseases associated with the nucleotide-binding oligomerization domain-containing protein 2 (NOD2) pathway and the interleukin (IL)-1β pathway leading to sustained cytokine release by innate cells. In genetic terms, both polygenic and monogenic autoinflammatory diseases have been described (4). A viral infection leading to hyperinflammation is not an autoinflammation, because auto-inflammation, by definition, requires a lack of infectious trigger. This is in contrast to pHLH, which is known to be triggered by viruses.

The release of granules containing cytolytic effector molecules by cytotoxic lymphocytes is not only essential in the host’s defense against viruses and other pathogens but also serves to terminate immune responses (5). The latter is evidenced by the development of systemic hyperinflammation in patients who lack perforin, the pore-forming molecule delivered to target cells during granule-mediated cytotoxicity (1, 2). This sepsis-like disease is characterized by a number of clinical and laboratory criteria that include fever, splenomegaly, bicytopenia, hemophagocytosis, and hyperferritinemia, among others. According to the current diagnostic criteria (HLH-2004), HLH is diagnosed in a patient when fulfilling the following criteria: either 1) a molecular diagnosis consistent with a mutation previously associated with HLH or 2) 5 of 8 of the following clinical parameters: fever; splenomegaly: bicytopenia, affecting at least two of three lineages in peripheral blood (hemoglobin<90g/L (in infants <4 weeks: hemoglobin <100 g/L), platelets < 100x10^9^/L, neutrophils <1.0x10^9^/L); hypertriglyceridemia (fast triglycerides >265mg/dl) and/or hypofibrinogenemia (fibrinogen ≤1.5g/L); hemophagocytosis in the bone marrow or spleen or lymph nodes; low or absent natural killer (NK) cell activity; high ferritin levels (≥500μg/L); high levels of soluble CD25 (≥2,400U/mL) (6).

HLH can also occur in individuals with germline mutations in genes not related to any defect in the cytotoxic machinery. Furthermore, the so called “acquired” forms of HLH develop concomitantly to other infectious, malignant, autoimmune and rheumatological diseases such as systemic juvenile idiopatic arthritis (sJIA) (7, 8). By contrast to pHLH, these forms have been also named secondary HLH (sHLH). In particular, sHLH associated with rheumatologic/autoimmune conditions is often named macrophage activation syndrome (MAS). pHLH and MAS share clinical symptoms but MAS lacks the familial link and/or genetic causative mutation.

HLH is a cytokine storm syndrome. pHLH and MAS cause similar clinical manifestations and share some (but not all) impairments in immune pathways. Episodes of pHLH and episodes of MAS can both be triggered by infections (9). The immune responses to the trigger become persistent and go out of control, which leads to multiorgan damage and a sustained hyperinflammatory response - the main dangers, rather than impaired clearance of the viral trigger. However, in some disorders with HLH (e.g. XLP-1), the initial viral infection is poorly controlled. The immune dysregulation in HLH leads to an exaggerated, prolonged immune activation. This prompts the cytotoxic cell to secrete larger amounts of cytokines (including IFNγ), which directly activate macrophages. The defects in granule-mediated cytotoxicity in HLH compromise the ability of NK cells and CD8+ T cells to kill their target cells. Perforin-deficient CD8+ T cells interact with APCs for longer than usual (10, 11). Defective disengagement between the cytotoxic cell and its target leads to repetitive calcium release in the cytotoxic lymphocyte and increased production of proinflammatory cytokines. IFNγ and TNFα (a) stimulate hemophagocytic activity in macrophages, (b) continuously activate T cells during antigen presentation, and (c) induce the production of other proinflammatory cytokines, leading to a cytokine storm. Macrophage-secreted cytokines (such as IL-1α, IL-1β, IL−6, IL1β and IL-18, in MAS) maintain the CD8^+^ T cells in an activated state and thus create a cytokine storm feedback loop.

Reduced fratricide killing furthermore contributes to the pathogenesis of HLH: during viral infections, NK cells have an immunoregulatory role by controlling overactivated CD4^+^ and CD8^+^ T cells (12, 13), a function which is disabled in the context of defective granule-dependent cytotoxicity.

The characterization of novel monogenic HLH disorders has revealed additional disease mechanisms. On one hand, APC resistance to killing may inhibit timely resolution of inflammation. Target cells themselves have an active role in determining susceptibility to granule-mediated cytotoxicity, through the cell-surface expression of cytotoxicity receptor ligands (14). On the other hand, cell-intrinsic overproduction of cytokines or dysregulated cytokine control may contribute to HLH. Systemic hyperinflammation can be triggered by inflammasome activation or by the dysregulation of cytokine mRNA transcription (15–18).

**Figure 1:**
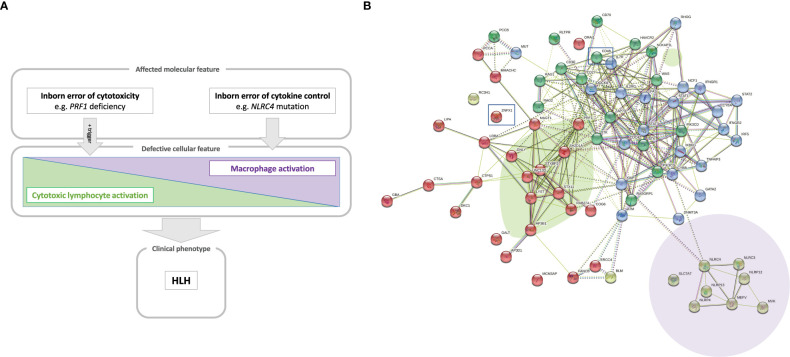
**(A)** A model of the hyperinflammatory spectrum, ranging from errors of cytotoxicity to lack of cytokine control with macrophage activation. From the molecular point of view, HLH-associated gene mutations can be divided into inborn errors of cytotoxicity and inborn errors of cytokine control (upper panel). These genetic defects have consequences on the cellular level on a spectrum ranging from impaired lymphocyte cytotoxicity to macrophage activation. A trigger (e.g. a viral infection) for HLH is commonly found in the case of impaired lymphocyte cytotoxicity (middle panel). Both entities, inborn errors of cytotoxicity and inborn errors of cytokine control, may converge clinically to HLH (lower panel). **(B)** Genetic determinants of HLH and hyperinflammatory diseases. HLH-associated genes identified by searching the literature for reported cases are shown with their known and predicted protein-protein interaction network linked graphically using the STRING database and algorithm (182). The green oval highlights genes whose products are involved in granule-mediated cytotoxicity. Genes whose products interact with *NLRC4* are highlighted by the mauve oval. HLH has also been described to occur in chronic granulomatous disease (genes marked in dark blue in the upper right corner) and (severe) combined immunodeficiency (genes in the upper left corner, such as IL2RG, ZAP70 and IL7RA). Two newly reported molecular causes, CD48 and ZNFX1, are highlighted (by squares), which we position here within the pathogenic pathways that lead to HLH.

Thus, HLH can occur in a whole array of clinical settings and so is considered as a group of inflammatory disorders. Whole-exome and whole-genome sequencing methods have immensely amplified the ability to identify novel, pathogenic gene variants causing IEI (19). We and others have used these technologies to discover novel genetic inflammatory syndromes with HLH (14, 15, 20–22). Here, we propose a model that positions a number of conventional and novel monogenic inflammatory disorders on the HLH spectrum that ranges from inborn errors of cytotoxicity to inborn errors of cytokine control – all of which converge clinically to HLH (**Figure 1A**).

## 2 Cytotoxic lymphocyte subsets and granule-mediated cytotoxicity

Cytotoxic lymphocytes correspond to various subsets of innate and adaptive cells that recognize and attack malignant, stressed or virally infected cells. These include innate NK cells, adaptive cytotoxic T lymphocytes (CTLs), and other less abundant unconventional T lymphocytes. NK cells and CTLs are both capable of death receptor activation and the release of granules containing cytolytic effector molecules. These cytotoxic cells also produce proinflammatory cytokines, such as interferon gamma (IFNγ). CTLs have a CD8 complexed T cell-receptor (TCR) that recognizes peptides derived from (for example) intracellular pathogens or malignant cells. The peptides are presented by major histocompatibility complex (MHC) class I molecules on APCs (23). Specific peptide recognition triggers CD8^+^ T cell activation, the acquisition of cytotoxic effector functions, and the development of the CTL memory program (24).

As an evasion strategy, some pathogens and malignant cells can downregulate MHC I expression. However, NK cells (the main innate lymphoid cell subset) complement CTLs by detecting and killing target cells that have downregulated their cell-surface expression of MHC class I. Furthermore, the cytotoxic activities of CTLs and NK cells have complementary time scales; whereas NK are innate cells that rapidly induce cell cytotoxicity, adaptive CTL responses require an antigen encounter and the development of effector status (25). As innate immune cells, NK cells lack specific, rearranged lymphocyte receptors and mount rapid, first-line effector responses against infected, malignant or stressed cells. They integrate signals of an array of germline-encoded HLA-specific and non-HLA-specific activating and inhibitory receptors. An NK cell combines and integrates the inputs of its various receptors, which fine-tunes its effector outcome (25, 26). For example, the lack of MHC class I ligands (detected by inhibiting receptors such as killer immunoglobulin (Ig)-like receptors and NKG2A) triggers cytotoxicity. The detection of ligands present in altered cells by activating receptors (e.g. NKG2D and natural cytotoxicity receptors) also promotes cytotoxicity. CD16 is another activating receptor present in the CD56^dim^ cytotoxic subset of NK cells; it binds the Fc portion of IgG antibodies in antibody-coated target cells and triggers antibody-dependent cell cytotoxicity (ADCC). Other activating and inhibitory receptors (including 2B4, CD2, DNAX accessory molecule-1 (DNAM-1) and T cell immunoglobulin and mucin domain-containing protein 3 (TIM-3)) help to fine-tune NK cell cytotoxicity and are discussed below because of their roles in immune regulation and hyperinflammation. Despite the NK cells’ clear role in innate immunity, it has recently been discovered that these cells also develop “memory-like” responses. Hence, NK cell subsets can expand, contract and later vigorously respond to previously encountered haptens, viral antigens and vaccines (27). This exciting new discovery has changed the paradigm for the link between immune memory and adaptive responses. However, the mechanisms that contribute to the NK cells’ immune memory have not yet been identified and characterized in detail (28).

Other less abundant cytotoxic cell subsets are important for immune regulation in hyperinflammatory diseases. Unconventional T cells (such as NKT cells, γδ T cells, invariant NKT (iNKT) cells, and mucosa-associated invariant T cells) share features of both NK and T cells. They recognize antigens other than classic peptides (such as microbial metabolites or lipid antigens) presented by non-polymorphic molecules other than MHC (such as CD1d and butyrophillins) and semi-invariant or γδ TCRs (29, 30). Peripherally induced regulatory T cells can also exert cytotoxicity towards myeloid APCs in a perforin-, granzyme B- and HLA class I-dependent manner, assisted by lymphocyte function-associated antigen 1, CD2-CD58, and CD226-CD155 (31, 32).

Granule-mediated cytotoxicity is the cytotoxic lymphocyte’s main killing mechanism. Upon activation, cytotoxic lymphocytes polarize secretory lysosomes that are anchored to microtubules and contain cytotoxicity effector molecules. The secretory lysosomes and the microtubule-organizing center move progressively towards the immune synapse formed between the killer cell and the target cell. Cytotoxic granules dock at the inner leaflet of the effector cell plasma membrane, near a cluster of TCRs (the central supramolecular activation cluster) and release their contents into the synaptic cleft *via* exocytosis (5). Perforin is a pore-forming protein present in the lymphocytes’ cytotoxic granules. It is released as a monomer but can form oligomeric pre-pores that can dock with (but do not insert into) the target membrane (33). Insertion into the target membrane occurs only upon a conformational change of the perforin, enabling polymerization and formation of 22-mer pores in the target cell membrane, which then facilitate the entry of soluble cytotoxic effectors into the target cell (34). Although both target and killer cell are exposed to perforin in the immune synapse, only the target cell membrane is disrupted. Indeed, two protective mechanisms prevent the CTL from being killed during the cytotoxicity response: (i) the formation of highly ordered lipid rafts, and (ii) the exposure of negatively charged phosphatidylserines, which inactivate residual perforin (35).

Granzymes are the main cytotoxic effectors in secretory granules. Of the various types of granzymes, granzymes A and B the most abundant and best characterized. Granzyme B engages caspase-dependent apoptosis of the target cell and cleave initiator pro-caspases, such as pro-caspase-3 and the pro-apoptotic molecule Bid. Granzyme A triggers caspase-independent cell death *via* the disruption of mitochondrial metabolism and the generation of reactive oxygen species, which enable the formation of the SET complex of nucleases. Upon translocation to the nucleus, Granzyme A cleaves and releases SET complex nucleases, which eventually leads to DNA damage (36). The less studied granzyme K is present in the immunoregulatory CD56^bright^ NK cell subset (37). The latter are also able to kill autologous activated T cells in a granzyme K dependent manner (38). Interestingly, CD56^bright^ NK cells expand during disorders of hyperinflammation and cytotoxic cell disturbance (14, 39). Perforin and granzymes have a synergistic effect on granule-mediated cell cytotoxicity. Granzymes A, K and M are differentially expressed on cytotoxic T and NK cell subsets (37). Data from experiments in granzyme-deficient mouse models indicate that the granzymes have redundant functions, in order to overcome viral evasion strategies (37, 40). Redundant, compensatory granzyme functions can mediate tumor rejection in granzyme-deficient mice (41). It is noteworthy that only mice lacking both granzymes A and B are susceptible to lymphocytic choriomeningitis virus (LCMV) and the poxvirus ectromelia but are still resistant to other viruses. In contrast, perforin has an essential, non-redundant role in cytotoxicity (41, 42). This might also be why granzyme deficiency has not (yet) been linked to HLH per se, given that most gene-hunting workflows in the field of IEI are based on a monogenic disease hypothesis.

Granulysin is another membrane-disrupting effector molecule present in cytotoxic granules of CTLs and NK cells. It primarily attacks cardiolipin-rich microbial cell membranes rather than cholesterol-rich mammalian cell membranes (43) but does appear to be somehow involved in human cell cytotoxicity (44). However, granulysin and has not been studied in the context of HLH.

In addition to granule-mediated cytotoxicity, cytotoxic cells can also induce target cell killing through death receptors. The main death receptors are Fas (CD95) and TNF-related apoptosis-inducing ligand (TRAIL) receptors. After ligand binding, Fas ligand and TRAIL transmembrane death receptors recruit Fas-associated protein with death domain (FADD) adapter proteins to their death receptor domain and activate caspase-dependent apoptosis (45). A small proportion of NK cells can kill several target cells consecutively. This process is known as “serial killing” and is important for the elimination of infected and malignant cells (46). During serial killing, cytotoxicity mechanisms are tightly regulated by NK cells. The first kills result from granule-mediated fast cytolysis, whereas later events switch to slower death receptor killing following upon a decrease in the granule count and the upregulation of FAS ligand (47). Both mechanisms are enhanced by cytokines like IL-2, IFNγ and tumor necrosis factor alpha (TNF-α), which also promote inflammation. The importance of FAS and FAS ligand for immune regulation is obvious in patients with autoimmune lymphoproliferative syndrome (ALPS) due to mutations in the *FAS* and *FASLG* genes (48). Although perforin deficiency is a fatal disorder, patients with ALPS easily reach adulthood.

Cell death caused by limited availability of growth factors, such as following the resolving of an immune response, is more dependent on the proapoptotic factor BCL-2 interacting mediator of cell death (BIM) (49, 50).This is independent of other death receptors, such as FAS. Therefore, BIM-dependent cell death has been implicated in controlling lymphocyte contraction following resolution of an immune response, where conditions of lower pro-inflammatory cytokines and growth factors are created. On the other side, during chronic infections, where antigen persists and lymphocyte expansion is promoted, lymphocyte expansion is mainly controlled by FAS-dependent cell death (51). BIM could also be implicated in the resolution of lymphoproliferation in ALPS (52). In humans, a common deletion polymorphism in BIM that enables the synthesis of an alternatively spliced isoform has been associated to low efficacy of tyrosine kinase inhibitors in cancer (53). In addition, mice experiments have shown a role of this protein in controlling autoimmunity, but also in controlling APCs expansion (54), therefore raising a possibility of a predisposition for HLH.

An additional indirect mechanism that cytotoxic lymphocytes use to promote killing is the production of proinflammatory cytokines, such as IFNγ and TNFα. IFNγ directly enhances the cytolytic activity of NK cells and CTLs (55). TNFα binds to TNF receptors 1 and 2 and can thus trigger cell death upon FADD adaptor recruitment or trigger pro-inflammation through nuclear factor kappa B activation (56). Lastly, another indirect mechanism is the competitive advantage given to activated CD8+ T cells by homeostatic cytokines such as IL-2, to the detriment of regulatory T cells (Tregs) in the context of HLH (57, 58).

## 3 HLH and HLH-like hyperinflammatory syndromes

HLH corresponds to a clinical phenotype with diverse triggers and disease mechanisms. A comprehensive overview of the functional networks of proteins encoded by genes reportedly linked to HLH is given in **Figure 1B**.

### 3.1 Primary HLH

Primary HLH (pHLH) corresponds to a group of disorders caused by IEI affecting genes, whose products are involved in granule-mediated cytotoxicity. The signs and symptoms of pHLH usually appear at an early age, although the disorder may also develop later in life. The clinical manifestations of pHLH include fever, hepatosplenomegaly, and multiorgan infiltration and damage (e.g. bone marrow failure and damage to the central nervous system) (59–61). The presence of tissue macrophages with hemophagocytic activity (referred to as histiocytes) is a hallmark of pHLH. Other observed clinical abnormalities include bicytopenia, hypercytokinemia, overactivated T lymphocytes, elevated ferritin levels, and elevated levels of soluble interleukin 2 (IL-2) receptor α (also referred to as soluble CD25 (sCD25)). Primary HLH is diagnosed when the patient meets at least five of the eight established clinical criteria (6) or has compatible molecular findings. The estimated incidence of pHLH is 1 per 3000 inpatients in tertiary care pediatric hospitals and 1 per 50000 newborns (59, 62). Primary HLH is a potentially fatal sepsis-like disease; for survival, immunosuppressive treatment and then hematopoietic stem cell transplantation (HSCT) are generally required (63). Episodes of hyperinflammation are caused by uncontrolled, excessive immune responses, mostly upon exposure to viral or bacterial triggers (9). However, a triggering event or infection cannot be found in all individuals with HLH, e.g. some patients with intrauterine HLH. Rather than being a direct effect of an infectious trigger, pHLH develops because of impaired regulation of inflammation and lacking termination of immune responses by the granule-mediated cytotoxicity pathway (64).

Primary HLH is caused by inherited pathogenic variants in genes involved in different stages of the perforin-dependent granule-mediated cytotoxic pathway. Familial HLH type 2 (FHL2) is caused by biallelic deleterious mutations in the gene coding for perforin (*PRF1*). To date, more than 120 different *PRF1* mutations have been described (65) and account for 20-50% of cases of pHLH. However, some mutations are found also in healthy older adults – sometimes even in their homozygous form (66). The perforin knock-out mouse infected with LCMV is the “gold standard” model of pHLH and has provided valuable information on the pathogenesis of this disorder (58, 64, 67, 68).

Other HLH-associated mutations affect genes whose products are involved in the docking, priming and membrane fusion of cytotoxic granules (64, 69, 70). Familial HLH (FHL) type 3 (FHL3), type 4 (FHL4) and type 5 (FHL5) patients show degranulation defects. FHL3 is caused by pathogenic variants in the *UNC13D* gene encoding the Munc13-4 protein involved in priming the secretory granules. *UNC13D* mutations account for 30-35% of pHLH cases, although the prevalence varies as a function of the ethnicity and the geographic area; for example, the prevalence of *UNC13D* mutations is higher in northern Europe (71). FHL4 patients present mutations in the syntaxin 11 gene (*STX11*) involved in the membrane fusion between the cytotoxic granule and the target cell (72). Mutations in the *STXBP2* gene (coding for syntaxin-binding protein 2, which assists membrane fusion in exocytosis) cause FHL5 (73). There is also a related group of disorders characterized by concomitant HLH and hypopigmentation. For instance, Griscelli syndrome type 2 is caused by pathogenic variants of the *RAB27A* gene; the encoded GTPase signaling protein is expressed in many (but not in the central nervous system (74)) and is involved in late granule exocytosis stages. Hence, *RAB27A* pathogenic gene variants affect not only cytotoxic granules but also melanosome degranulation. Some patients also present neuropathy associated with the sequelae of HLH (75). Chediak-Higashi syndrome is another rare disease associated with hypopigmentation, HLH, impaired cytotoxicity, and the presence of enlarged lysosomal structures in cells and hair shafts. In this syndrome, pathogenic gene variants of *LYST* (coding for a lysosomal traffic regulator) impair the release of cytotoxic granules into the immune synapse. Lysosomal trafficking is an important process in neurons. Some *LYST* mutations are associated with neuronal affectations. Moreover, a mouse model bearing a mutation in the LYST protein’s conserved WD40 domain shows a neurodegenerative phenotype with Purkinje cell loss, rather than alterations in the immune system (76). Furthermore, patients with Chediak-Higashi syndrome may develop a neurodegenerative disease marked by cerebellar ataxia and peripheral neuropathy – even after successful HSCT (77). Hermansky-Pudlak syndrome 2 is another rare, multisystem disorder associated with HLH. It is caused by *AP3B1* pathogenic gene variants that affect lysosomal protein sorting and lead to cytotoxic lymphocyte defects in patients. A summary of the genetic, epidemiological, clinical and immunological characteristics of pHLH, together with secondary and novel forms of HLH are compiled in **Table 1**.

**Table 1:** Conventional and novel HLH, HLH-like and HLH-associated disorders: genetics, epidemiology, clinical manifestations, and immune dysregulations.

Disease	Gene symbol	Protein name (s)	Cellular expression	Impaired function	Clinical manifestations and concomitant diseases	Trigger	Mouse model
Primary HLH (FHL1)	Unknown	Unknown	Unknown	Cytotoxicity	HLH	Intracellular infections (viruses, bacteria, parasites	Not described
Primary HLH (FHL2)	*PRF1*	Perforin-1 (PERF)	Cytotoxic lymphocytes	Cytotoxicity	HLH	Viruses (EBV, CMV, other herpesviruses, parvovirus B19, adenoviruses)	Perforin-deficient mice infected with LCMV
Primary HLH (FHL3)	*UNC13D*	Protein unc-13 homolog D (Munc13-4)	Leukocytes, lung, placenta	Degranulation, cytotoxicity	HLH	Viruses (EBV, CMV, other herpesviruses, parvovirus B19, adenoviruses)	*Unc13d* mutated “jinx” mice infected with LCMV
Primary HLH (FHL4)	*STX11*	Synthaxin-11 (STX11)	Cytotoxic lymphocytes	Degranulation, cytotoxicity	HLH	Viruses (EBV, CMV, other herpesviruses, parvovirus B19, adenoviruses)	*Stx11*-deficient mice infected with LCMV
Primary HLH (FHL5)	*STXBP2*	Syntaxin-binding protein 2 (STXBP2, Munc18-2)	Cytotoxic lymphocytes, intestinal and renal epithelium	Degranulation, cytotoxicity	HLH	Viruses (EBV, CMV, other herpesviruses, parvovirus B19, adenoviruses)	Not described
Griscelli syndrome type 2	*RAB27A*	Ras-related protein Rab-27-A (RAB27A	Leukocytes and melanocytes	Degranulation, cytotoxicity	HLH. Concomitant hypopigmentation.	Viruses (EBV, CMV, other herpesviruses, parvovirus B19, adenoviruses)	*Rab27a*-deficient *ashen* mice infected with LCMV
Chediak-Higashi syndrome	*LYST*	Lysosomal-trafficking regulator (LYST)	Leukocytes, melanocytes and neurons	Lysosome trafficking, cytotoxicity	HLH. Concomitant hypopigmentation.	Viruses (EBV, CMV, other herpesviruses, parvovirus B19, adenoviruses)	Lyst mutated mice (*souris* strain) infected with LCMV
Hermansky-Pudlak syndrome type 2 (HPS2)	*AP3B1*	Adaptor protein complex 3 (AP-3) complex subunit beta 1 (AP3B1)	Multiple cell types, including melanocytes, fibroblasts, platelets, and monocytes.	Lysosome storage and trafficking, cytotoxicity	Multisystem disease. Concomitant hypopigmentation, bleeding.	Viruses (EBV, CMV, other herpesviruses, parvovirus B19, adenoviruses)	*Ap3b1*-deficient *pearl* mice infected with LCMV
Secondary HLH (MAS)	*-*	–	–	Regulation of hyperinflammation/diverse	HLH. Concomitant rheumatic, inflammatory, malignant or infectious diseases.	Underlying autoimmune or autoinflammatory disease, lymphoma, viral infection (herpes viruses, HIV, influenza), bacterial (mycobacteria), fungal and parasitic infections	Toll-like receptor 9 stimulation in wild-type and IL-6 transgenic mice or in combination with IL-10 receptor-blocking antibody. Infection with *Salmonella* or *Trypanosoma* in wild-type mice. Humanized mouse models with transfer of the patient’s immune cells to immunodeficient mice.
Secondary HLH (MAS)	*NLRC4*	NLR Family CARD Domain Containing 4	Leukocytes, higher expression in macrophages	Inflammasome activation	HLH	Inflammasome	*Nlrc4* mutant mice, with or without cold exposure
XLP-1	*SH2D1A*	SH2 domain-containing protein 1A (SAP)	Cytotoxic lymphocytes	Signaling triggering cytotoxicity	HLH. Concomitant hypogammaglobulinemia, lymphoma.	EBV, occasionally other viruses	*Sh2d1a*-deficient mice infected with LCMV or MHV-68
HLH	*HAVCR2*	Hepatitis A virus cellular receptor 2 (TIM-3)	T lymphocytes and other immune cells	Checkpoint inhibitor regulation	HLH. Concomitant subcutaneous panniculitis-like T cell lymphoma.	Not described	Not described
HLH and autoinflammation	*NCKAP1L*	NCK associated protein 1 like	Hematopoietic cells	Cytoskeleton regulation, T cell homeostasis	HLH, autoinflammation, neonatal pancytopenia	Not described	Not described
HLH and hyperinflammation	*RC3H1*	Roquin-1	Leukocytes	mRNA post-transcriptional regulation on immune genes	Hyperinflammation	Not described	Toll-like receptor 9 stimulation in sanroque mice
NOCARH	*CDC42*	Cell division cycle 42, isoform 1	Ubiquitously expressed	Cytoskeleton rearrangement, migration and cell proliferation	Neonatal onset of pancytopenia, autoinflammation, rash and HLH	Not described	Not described
Recurrent hyperinflammation	*CD48*	CD48	Immune cells	Signaling triggering cytotoxicity	HLH-like	Not described	*Cd48*-deficient mice infected with LCMV
XLP-2	*BIRC4*	XIAP	Ubiquitously expressed	Apoptosis, inflammation signaling, inflammatory cell death,	HLH. Concomitant hypogammaglobulinemia, colitis.	EBV, occasionally other viruses	*Birc4*-deficient mice infected with MHV-68
Multisystem inflammation, vulnerability to infections	*ZNFX1*	NFX1-*type zinc finger-containing* protein *1*.	Ubiquitously expressed, greater expression in hematopoietic tissue	IFN-dependent transcript regulation	HLH and HLH-like	Viral and mycobacterial infections	Not described
HLH (occasionally)	*STAT1*	STAT1	Ubiquitously expressed, greater expression on leukocytes	IFN-dependent signaling	HLH	*Mycobacterium bovis* (1 case)	Multiorgan immune infiltration and hypercytokinemia in *Stat1*-deficient mice infected with LCMV (although not described as an HLH model)

CMV, cytomegalovirus; EBV, Epstein-Barr virus; FHL, familial hemophagocytic lymphohistiocytosis; HLH, hemophagocytic lymphohistiocytosis; LCMV, lymphocytic choriomeningitis virus; MAS, macrophage activation síndrome; MHV-68, murine gammaherpesvirus 68; NFX, nuclear transcription factor X-box binding; NOCARH, neonatal onset of pancytopenia, autoinflammation, rash, and episodes of hemophagocytic lymphohistiocytosis; STAT, signal transducer and activator of transcription; XIAP, X-linked inhibitor of apoptosis; XLP, X-linked lymphoproliferative syndrome.

### 3.2 X-linked lymphoproliferative disease

X-linked lymphoproliferative disease (XLP-1) is a life-threatening lymphoproliferative disorder that arises in male patients with mutations in the X-linked *SH2D1A* gene (78, 79). The incidence of XLP-1 is 1 to 3 per million males, and 45-70% of patients with XLP-1 develop HLH (78, 79). Patients with XLP-1 have an impaired ability to clear EBV infections. The seroprevalence of EBV in adults is 90%. Diseases caused by EBV are mild in children, moderate-to-severe in teenagers and immunocompetent adults but life-threatening in patients with *SH2D1A* mutations; the survival rate in the latter is 20%, and the disease features lymphoproliferation, multiple organ infiltration and multiple organ failure (80). 25-30% of patients with XLP-1 develop B-cell lymphoma associated with EBV infection (80). In addition, 35% of patients have not been exposed to EBV but are diagnosed because of their family medical history (81). At the time of writing, 100 patients with XLP-1 have been described in the literature (82). It is not completely understood how viral infections trigger HLH episodes in susceptible individuals. Putative mechanisms include the direct interference of antiviral responses with cytokine balances, the direct infection of cytotoxic cells or other key cells in HLH, disturbance of immune homeostasis, the capacity of viruses to encode anti-apoptotic proteins delaying the apoptosis of infected immune cells or the chronic stimulation of pattern recognition receptors. Other viral evasion strategies that might promote HLH are the downregulation of MHC class I on NK cells or the suppression of the cytotoxic function of NK cells by downregulating the expression of perforin and SAP or encoding Fc receptors that block viral-specific antibodies diminishing antibody-dependent cell cytotoxicity (9).

*SH2D1A* encodes SAP, a small intracellular molecule belonging to a family of adaptors containing a Src homology 2 domain and a short C-terminal tail, that includes Ewing’s sarcoma-activated transcript-2 (EAT-2) and EAT-2-related transduced. SAP binds to phosphorylated immunoreceptor tyrosine-based “switch” motifs (ITSM) of the SLAM family (SLAMF) of receptors within the CD2 family of leukocyte surface receptors, triggering lymphocyte cytotoxicity upon recruitment of the tyrosine kinase FynT and also preventing the binding of other inhibitory phosphatases (83, 84). The impediment to bind SAP to 2B4 SLAMF receptor enables the binding of other inhibitory proteases to intracellular 2B4 ITSMs, triggering the receptor’s inhibitory function rather than the SAP-mediated activating signal (85). Various *SH2D1A* mutations have been described as affecting the binding to interactants (such as SLAMF receptors and FynT) or decreasing the half-life of the SAP protein (86, 87). Along with the molecular identification of SAP mutations, other methods for the rapid diagnosis of XLP-1 have been suggested. However, the measurement of intracellular SAP expression might not be relevant for mutations affecting SAP’s function or half-life.

SAP pathogenic gene variants lead to a reduction in NK cell cytotoxic activity (79, 88–91). Patients with XLP-1 have abnormally low levels of NK and CD8+ T cell cytolytic activity towards EBV-infected B cells. A rapid screen that combines intracellular SAP expression and a 2B4-directed reverse ADCC (R-ADCC) assay of murine Fc receptor-expressing target cells has shown promising results (92). In this study, SAP^-^ and SAP^+^ NK clones from healthy female heterozygous carriers of SAP mutations provided information on the molecular defects in SAP deficiency. SAP^-^ clones showed low cytotoxic activity towards CD48^+^ target cells in an R-ADCC assay triggered by 2B4 crosslinking. In contrast, SAP^+^ NK clones exerted cytotoxicity upon 2B4 crosslinking, giving an overall neutral response at a polyclonal level in bulk populations (92). Patients with XLP-1 have defective NK cell cytotoxicity towards SLAMF-expressing hematopoietic cells. Moreover, SAP-deficient mice and patients with XLP-1 display enhanced NK responses to non-hematopoietic cells (93). This difference has been attributed to a defect in NK cell education (a mechanism for fine-tuning the NK cells’ sensitivity to activating and inhibitory signals) in XLP-1 (94). Signaling during NK cell education is mediated by SLAMF6 and depends on SAP, which blocks the binding of the inhibitor phosphatase SHP1 to the SLAMF receptor. As mentioned before, SAP not only mediates SLAMF-activated signaling but also prevents inhibitory SH2-containing proteases from binding to SLAMF receptors (84, 95). Natural cytotoxicity receptors (such as NKp46 and CD16) have compensatory activity in patients with XLP-1 (93). SAP is crucial for the development of NKT cells because the latter are absent in patients with XLP-1 and in SAP-deficient mice (78, 96). Mice with a SAP mutation that abolishes the recruitment of Fyn have a defect in NKT cell development in the thymus and a lack of invariant NKT (iNKT) cells (97). NKT developmental defects have been found in mice with mutations in all SLAMF receptors (98–100). NKT cells are also absent/very reduced in patients. For instance, NKT cells showed undetectable levels with α-galactosylceramide-loaded CD1d tetramers and/or antibodies targeting the invariant Vα24 Vβ11 TCR, when compared with controls or patients with other IEI (101). Thus, it has been suggested that low/absent NKT cell frequencies could be a diagnostic parameter for XLP1. However, data from other studies indicate that the frequency of NKT cells in adult and pediatric XLP1 patients is variable and point against the use of NKT cell levels to exclude a diagnosis of XLP1 (102). In SAP-deficient patients, high levels of T cell proliferation have been observed upon viral infection, despite the inability to eliminate virus-infected cells or to produce IFNγ. In mice, T cells proliferate and survive the acute infection (88). SAP-deficient mice have problems controlling chronic LCMV infections, due to defects in humoral response (89). These defects can be rescued by reconstitution with wild-type CD4^+^ T cells (103). Patients with XLP-1 also have profound defects in the humoral response. 50% of patients with XLP-1 have hypogammaglobulinemia (82). Defective T follicular helper (TFH) and T helper 2 (Th2) cell development and defective Th2 cytokine production are also observed (88, 89, 91, 104). SAP-deficient mice have a defect in TFH cell development in the germinal centers. The TFH cell type is crucial for the generation of high-affinity antibody responses (78). In the absence of SAP, SLAM family receptor signaling to TFH is inhibitory, since mice with mutations in all the SLAM family receptors do not have a defect in TFH development (98). XLP1 patients also display a deficiency in the formation of germinal centers in the spleen (105). In tune with this defect, patients show a reduction of germinal center-dependent isotype-switched CD27+ memory B cells in peripheral blood. However, despite this reduction, the few patient’s IgM+ CD27+ B lymphocytes present in circulation have the capacity to undergo somatic hypermutation (105).

The XLP-1-related disorder XLP-2 has been linked to a deficiency of X-linked inhibitor of apoptosis (XIAP) protein, caused by pathogenic gene variants in the *XIAP*/*BIRC4* gene. More than 90 such mutations have been described since 2006 (106, 107). XIAP deficiency has similarities with SAP deficiency, such as EBV-triggered HLH, splenomegaly, cytopenia and hypogammaglobulinemia. However, patients with XLP-2 do not suffer from lymphoma; Other inflammatory symptoms (such as colitis, hepatitis, uveitis and arthritis) are considered a hallmark of XLP-2 (81). The anti-apoptotic protein XIAP is involved in innate immune signaling of pattern recognition receptors like dectin-1 and nucleotide oligomerization domain (NOD)-like receptors and also the regulation of the NOD-, LRR- and pyrin domain-containing protein 3 (NLRP3) inflammasome (106). Indeed, a functional assay demonstrating the XIAP-deficient monocytes’ inability to produce TNFα upon NOD2 stimulation had been established as a diagnostic tool (107, 108). XIAP also regulates adaptive immune functions such as activation-induced cell death (AICD), an important homeostatic mechanism for controlling and limiting activated T cells in conventional and unconventional T cells. In XLP-2 patients, AICD is also impaired in T cells (106). In contrast to XLP-1, patients with XLP-2 have a normal iNKT cell compartment (106). Regarding the humoral compartment, one third of XLP-2 patients experience mild hypogammaglobulinemia but the switched B cell compartment is not affected (106).

### 3.3 Secondary HLH or MAS

The term sHLH (or sometimes “sporadic” or “acquired” HLH) has generally been used to describe patients with (i) a disease meeting the clinical criteria for a diagnosis of HLH, (ii) none of the genetic defects mentioned in the above section on pHLH, and (iii) no XLP-1/2 syndrome. In addition and as later discussed, sHLH can also occur in patients with other IEI not related to cytotoxicity defects. Most patients with sHLH suffer from an inherited or acquired underlying disease or are receiving treatment that predisposes them to immune dysregulation. Secondary HLH can occur in autoinflammatory syndromes and is most frequently reported in systemic juvenile idiopathic arthritis (sJIA). Many rheumatologists prefer to use the term macrophage activation syndrome (MAS), rather than sHLH (8, 109–111) MAS appears later in life than pHLH but may also be life-threatening. pHLH and MAS have similar clinical manifestations. Although patients with pHLH have a permanent impairment in the granule-mediated cytotoxic activity of T lymphocytes and NK cells, there might be no impairment of granule-mediated cytotoxicity in patients with MAS or the impairment is only partial or transient. Animal models of MAS have been established by stimulating toll-like receptors in wildtype and IL-6 transgenic mice; these experiments highlighted the importance of IFNγ in MAS, while lymphocytes were not required for the induction of the disease, although critical to induce maximal disease (112–114).

In contrast to pHLH, inflammasome activation and IL-18 hypercytokinemia are the hallmarks of MAS pathogenesis in patients with *NLRC4* pathogenic gene variants. While impaired lymphocyte cytotoxicity is essential for a diagnosis of pHLH, intrinsically activated macrophages are essential for MAS in NLRC4-mutated patients. Furthermore, new mutations in the inflammasome-associated gene *NLRC4* reportedly cause some of the features of MAS (16, 17). We suggest a model in which NLRC4- and perforin pathogenic gene variants represent the two prototypes in this hyperinflammatory spectrum (**Figure 1A**). NLRC4 triggers the formations of inflammasomes, i.e. multimeric complexes assembled after stimulation by pathogens and that lead to the production of IL-1β and IL-18 and pyroptosis cell death after caspase-1 activation. Indeed, IL-18 hypercytokinemia has been linked to MAS in patients with sJIA and in animal models (115, 116). CD163, a scavenger receptor associated with phagocytosis in macrophages, is also a biomarker associated with MAS and EBV-triggered HLH (117). Strikingly, the A91V *PRF1* variant and mutations in *UNC13D* are more prevalent in patients with sJIA who developed MAS (118, 119). MAS is the most severe complication of sJIA and adult-onset Still’s disease and is also observed in patients with systemic lupus erythematosus. sJIA is a chronic, autoinflammatory disease of childhood characterized by the clinical features arthritis, fever, rash, swollen lymph nodes, hepatomegaly, and serositis. MAS-associated symptoms are observable clinically in 10% of sJIA cases and subclinically in 30-40% of cases. Nonsteroidal anti-inflammatory drugs constitute the first-line treatment, and corticosteroids and methotrexate are used as a second-line treatments. In treatment-resistant cases and in patients with sHLH, HSCT and monoclonal antibodies against the pathogenic proinflammatory cytokines are used. However, the treatment of sJIA has not been standardized.

In addition to MAS, patients infected with intracellular pathogens and patients being treated with immunomodulatory monoclonal antibodies or chimeric antigen receptor T cells may develop a cytokine release syndrome that resembles HLH. Secondary HLH can also develop in patients with inborn errors of metabolism, such as lysinuric protein intolerance, multiple sulfatase deficiency, galactosemia, Gaucher disease, Pearson disease, galactosialidosis, propionic acidemia, Niemann-Pick disease, and congenital disorders of glycosylation (120, 121).

### 3.4 Novel genetic inflammatory syndromes with HLH

The area of cell-cell contact between the cytotoxic lymphocyte and its target cell, the so called immunological synapse, is a highly organized area involving cytoskeletal rearrangement. Efficient killing by T cells and NK cells requires function of the actin cytoskeleton. Several mutations in genes with a role in active cytoskeleton remodeling have been associated with HLH. Indeed, pathogenic gene variants in *WAS*, *DOCK8* and *NCKAP1L* genes coding for cytoskeleton regulators in immune cells cause hyperinflammatory disorders with HLH hyperinflammation (20). Another novel HLH disorder associated with hematological impairments and features of autoinflammation was recently identified in five patients with a unique *de novo* missense mutation in the cell division cycle 42 (*CDC42*) gene (22, 122). This one specific mutation was associated with neonatal onset of cytopenia, autoinflammation, rash, and episodes of HLH (NOCARH). *CDC42* is a Ras-homologous (Rho) signaling GTPase protein involved in cytoskeleton rearrangement and cell migration. The p.R186C missense mutation identified affected the subcellular localization of the protein -aberrantly concentrated in the Golgi apparatus-, cell polarity, migration, proliferation and signaling. It profoundly affected hematopoiesis and compromised the normal composition and migration of bone marrow cells. This is a multisystem inflammatory disease with a strong autoinflammatory component, with characteristics similar to NLRC4 inflammosopathies. The autoinflammatory symptoms were attributed to the high spontaneous release of IL-18 by mononuclear cells from the bone marrow and the high IL-1β levels. In addition, the 4 identified patients developed HLH, lethal in all the patients but one, that survived upon anti-IFNγ treatment with emapalumab and HSCT. Indeed, IL-18 is a co-stimulatory factor for IFNγ production. An additional mechanism contributing to HLH in this novel hyperinflammatory syndrome was the defect in the NK cell capacity to form conjugates and to migrate, that diminished its cytotoxic potential (111, 116, 123). Differently than the NOCARH and HLH responsive to anti-IFNγ caused by the p.R186C mutation in the *CDC42* gene, novel additional mutations in *CDC42* C-terminus region have been recently associated to a clinical autoinflammatory syndrome responsive to IL-1 inhibitors (124).

Over the last few years, the establishment of novel associations between inborn errors of immunity (IEI) and HLH has broadened to beyond genes involved in cytotoxicity, cytoskeleton reorganization, and inflammasome activation, but also to the ones involved in checkpoint control, receptor signaling, and mRNA regulation. For instance, a novel association between HLH and loss-of-function mutations in the *HAVCR2* gene (coding for T cell immunoglobulin and mucin domain-containing protein 3 (TIM-3)) was identified in patients with subcutaneous panniculitis-like T cell lymphoma with associated HLH. TIM-3 is an inhibitory molecule expressed by T lymphocytes and other immune cells (21, 125). The TIM-3 negative checkpoint is a critical regulator of innate immunity and inflammatory responses and suppresses effector T cells by decreasing IFNγ-driven inflammation. The predisposition to HLH in individuals with defective TIM-3 function might therefore be explained by a defect in downregulating the T-cell response to IFNγ. Importantly, TIM-3 also regulates monocyte/macrophage activation. Thus, it is possible that TIM-3 deficiency leads in multiple ways to an increased inflammatory response and thereby to HLH.

Lymphocyte cytotoxicity is triggered upon contact with a target cell if sufficient activating signals are received. This implies that target cells are not passive bystanders during granule-dependent cytotoxicity. Indeed, we could gain insight in the role of resistance to cytotoxicity in the pathogenesis of HLH by studying a patient with a novel IEI affecting the 2B4-CD48 interaction. 2B4 (CD244) is a SLAMF receptor that signals through SAP and is crucial for controlling CTL responses to EBV (126). 2B4 is the SLAMF receptor with greatest number of ITSM SAP-binding motifs and is also the only heterotypic SLAMF receptor. Thus, 2B4 is not a self-ligand, but it interacts with CD48. In mice, signaling through 2B4 can be either costimulatory or coinhibitory, whereas there is evidence of a predominantly activating role in human subjects (127, 128). We recently identified a novel hyperinflammatory disorder with HLH caused by a *de novo* heterozygous mutation in the *CD48* gene (CD48^S220Yhet^) (14). This disorder is characterized by recurrent episodes of hyperinflammation, rash and IL-6 hypercytokinemia, while only moderately elevated sCD25 levels. A similar inflammatory pattern was triggered by LCMV infection in CD48+/- and CD48-/- mice (14). CD48 is expressed by almost all leukocytes (except for some long-term hematopoietic stem cell precursors) and functions mainly as a co-stimulatory and adhesion molecule (129). The CD48^S220^ residue is essential for the protein’s subcellular localization and serves as the attachment site for a glycosylphosphatidylinositol cell surface anchor. The S220Y pathogenic gene variant is associated with lower cell-surface CD48 expression and a lower cytotoxic ability for NK cells. The diminished expression of CD48 appears to be involved in a novel mechanism that contributes to hyperinflammation; target cells are less susceptible to killing and CD48-haploinsufficient immune cells are more resistant to elimination by granule-mediated cytotoxicity. Along with these functional alterations, maturation defects were also observed in cytotoxic lymphocytes. Thus, target cells’ resistance mechanisms to cytotoxicity can significantly contribute to immune dysregulation.

A novel homozygous mutation in the *RC3H1* gene (coding for roquin-1, a post-transcriptional regulator of mRNAs involved in immune responses) led to HLH and hyperinflammation in a patient and in a mouse model (18).

Signal transducer and activator of transcription 1 (STAT1) mediates both type I and type II IFN responses. Patients with a gain-of-function (GOF) mutation in *STAT1* can occasionally present HLH, despite the absence of IFNγ hypercytokinemia (130, 131). Conversely, *Stat1* knock-out mice develop multiorgan immune infiltration and hypercytokinemia upon LCMV infection (132). This data seem to be paradoxical. In the experimental *Stat1* knock-out mice model challenged with LCMV, the lethal multi-organic infiltration is dependent on highly expanded antigen-specific CD4+ T cells. In the clinical setting, *STAT1* GOF mutations have been linked to a permanent phosphorylated status of the transcription factor due to an impaired dephosphorylation and are associated to a broad clinical spectrum, from infection susceptibility to autoimmune manifestations, this later probably due to a strong type I IFN signaling mediated by the hyperphosphorylated STAT1 (131). In the clinical case with *STAT1* GOF linked to HLH, the hyperphosphorylated status of STAT1 was associated with a persistent overactivity of APCs, previously activated by innate immune receptors in the context of an infection (130). Interestingly, deficiencies in STAT2 and IRF9, the other two components that together with STAT1 form the heterotrimeric complex named Interferon Stimulated Gene Factor 3 (ISGF3), induce a prolonged type I IFN response due to lack of negative feedback of the IFN receptor (133). STAT2 and IRF9 mutations have been also recently linked to HLH episodes (134, 135).

ZNFX1 (NFX1-type zinc-finger-containing 1) is a highly conserved IFN-stimulated dsRNA sensor that restricts the replication of RNA viruses in mice and contributes to transgenerational inheritance in *C. elegans*, by binding to mRNA complexed to short non-coding RNAs (15, 136, 137). In humans, homozygous ZNFX1 destabilizing pathogenic gene variants were associated with multisystem inflammation, including HLH, monocytosis, and a predisposition to viral infections and mycobacterial disease (15, 138). Recent studies on patients suffering from COVID19 propose an anti-SARS-COV-2 role for ZNFX1 and other ZNF proteins, where ZNF protein activity positively correlated with the abundance of multiple immune cells implying an effective antiviral response (139). While the underlying mechanisms still need further investigation, the role of ZNFX1 in immune regulation highlights the importance of time and context in tuning innate response, to allow for proper elimination of viral material while preventing hyper-inflammatory responses. With regards to the spatial context, ZNFX1 has been shown to localize to liquid-like perinuclear condensates in *C.elegans* germ cells (136, 137), to stress granules of virally or chemically stressed cells (138), and to the proximity of the outer mitochondrial membrane in steady-state (137). We have shown that in the absence of ZNFX1, the half-life of the mRNA of interferon sensitive genes is prolonged (15). We therefore propose a mechanism whereby the helicase function of ZNFX1 is needed to remove mRNAs which has been formed in consequence of a viral infection. In the absence of ZNFX1, the mRNA of interferon sensitive genes remains more stable, allowing it to be translated again instead of being degraded. This prevents a return to homeostasis leading to the described hyperinflammatory syndrome. Overall, ZNFX1 plays a central role at both the very early and late stages of nucleic acids driven interferon responses, by regulating sensing and the return to homeostasis. As a consequence, patients with homozygous ZNFX1 destabilizing mutations suffered from multisystem inflammation, including HLH, and a predisposition to viral infections (118).

The molecular changes caused by inborn errors of granule-mediated cytotoxicity or cytokine control contribute variably to cellular impairments (such as impaired lymphocyte cytotoxicity or macrophage activation) that lead to uncontrolled hyperinflammation. Thus, the pathophysiological spectrum of HLH-associated disorders ranges from impaired lymphocyte cytotoxicity to macrophage activation (**Figure 1A**).

## 4 HLH-like manifestations in other immune-mediated diseases

### 4.1 Inborn errors of immunity

As described above, biallelic *PRF1* pathogenic gene variants cause pHLH. However, *PRF1* mutations concomitant to other IEI have been also described. ALPS is an immune dysregulation disorder that causes splenomegaly, lymphadenopathy, autoimmunity, susceptibility to lymphoma, and blood accumulation of double-negative CD4^-^ CD8^-^ T cells. Dianzani autoimmune lymphoproliferative disease (DALD) is a variant of ALPS that lacks the expansion of double-negative CD4^-^ CD8^-^ T lymphocytes (140). In most cases, ALPS is caused by a genetic mutation related to the FAS-mediated pathway of apoptosis. More than 70% of the mutations affect the *FAS* gene directly but mutations in *FASLG*, *CASP10*, *CASP8* and other genes have also been observed (48). A combination of a heterozygous *PRF1* mutation and a *FAS* mutation was identified in an ALPS patient with aggressive lymphoma (141). A larger study found that 2 of 14 ALPS patients and 6 of 28 DALD patients had an FHL-associated *PRF1* mutation, leading to diminished NK cell activity (121). Perforin-dependent activation-induced cell death operates as a compensatory mechanism in FAS-deficient T cells from ALPS patients (142). Mutations in the FHL3-associated *UNC13D* gene have been detected in six ALPS/DALD patients; although the patients’ NK cells showed normal levels of activity, granule exocytosis release was impaired in transfected cell lines (143). Furthermore, a SAP polymorphism affecting a key methylation site for the protein’s expression was significantly more frequent in ALPS/DALD patients than in controls (144).

A multicenter analysis identified 63 patients with other IEI meeting the diagnostic criteria for HLH (6). In a systematic evaluation of the patients’ clinical and immunological features, 30 had combined immunodeficiencies (including 12 with severe combined immunodeficiencies (SCIDs)) and 22 had chronic granulomatous disease (CGD) (145). 80% of patients with other IEI and who met the diagnostic criteria for HLH had either SCID or CGD. Although this study did not cover all known IEI, it is noteworthy that SCID and CGD were markedly over-represented because they account for only 15% of IEI overall. The remaining 20% (i.e. those not meeting the diagnostic criteria for HLH) included two patients with ALPS due to *FAS* mutations. In 79% of the affected cases, the HLH episode was associated with an infectious trigger. When comparing the subgroups with regard to their immunological and clinical variables, the serum level of sCD25 was lower in patients with T cell-dependent IEI than in patients with FHL. Thus, the study of other IEI associated with HLH provides data on the respective contributions of leukocyte subsets to hyperinflammatory diseases. In addition to rare complications of CGD and SCID (146, 147), rare cases of HLH have been also reported among patients with DiGeorge syndrome and Wiskott-Aldrich syndrome (148, 149).

### 4.2 The role of perforin in immune system cancers and bone marrow diseases

Inherited *PRF1* pathogenic gene variants cause FHL in early childhood. However, compound heterozygous missense *PRF1* mutations that do not fully abrogate perforin activity may have effects later in life (150). Granule-mediated cell cytotoxicity is a crucial mechanism for killing tumor cells. One study identified biallelic *PRF1* mutations in 4 out of 29 patients with primary lymphoma (151). Another study showed that 50% of cases of late-onset of FHL presented with lymphoma or leukemia. Interestingly, the mutations were not fully deleterious but caused protein misfolding that could be restored by a permissive protein folding temperature in *in vitro* assays (150). Missense mutations are also linked to a predisposition to cancer (152). *PRF1* mutations and decreased NK cell cytotoxic activity have been observed in patients with acquired aplastic anemia (a form of bone marrow failure) (153).

## 5 Novel insights into pathogenesis

**Figure 2:**
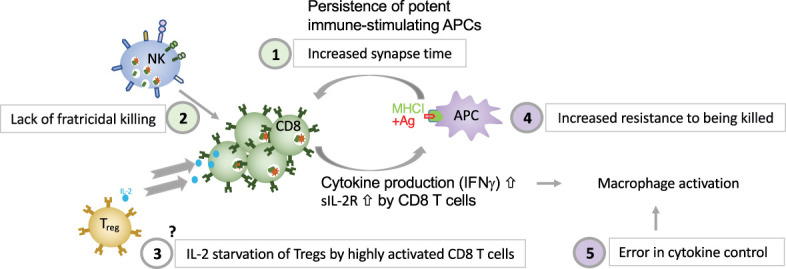
Five major disease mechanisms linking defective cytotoxicity and error in cytokine control with HLH (1). reduced clearance of triggering intracellular pathogens within APCs, which leads to an increase in the CTL-APC synapse time and repetitive Ca2+ release within (and thus activation of) the CTL (11); **(2)** defective NK-cell- and CTL-mediated immune regulation by fratricidal killing (12, 155), **(3)** excessive consumption of IL-2 by CTLs, which deprives Tregs, postulated by Humblet-Baron et al. (58), **(4)** persistence of immunostimulatory APCs (either due to increased resistance of APCs to killing as shown in a patient with CD48 deficiency (14) or due to persistence of immunostimulatory APCs due to CTL defect as demonstrated by Terrell & Jordan (154) in a murine model, and **(5)** error in cytokine control leading to macrophage activation.

Different dysregulated pathways contribute to HLH development. Deficient perforin-granule-dependent cytotoxicity leads to (i) reduced clearance of triggering intracellular pathogens within APCs, which leads to an increase in the CTL-APC synapse time and repetitive Ca^2+^ release within (and thus activation of) the CTL (11) and (ii) defective fratricidal killing (12); both resulting in excessive cytokine production by CTLs. Tregs are crucial regulators of immune responses and express high levels of CD25, the α subunit of the high affinity receptor for IL-2. The growth factor IL-2 is essential for T lymphocytes. A Treg dysfunction has been postulated in both patients with pHLH and in experimental models of pHLH, due to the preferential consumption of IL-2 by abundant, highly activated CD25^high^ CD8^+^ T cells (58). The precise role of Tregs in HLH needs further characterization. An important feedback loop which is critical for immune homeostasis and which fails in perforin-deficiency is the elimination of antigen-presenting dendritic cells. In a murine model of HLH has been shown that the persistence of potent, immunostimulatory dendritic cells, contributes to the pathogenesis (154), thus demonstrating a reciprocal relationship between perforin in CTLs and APCs’ function. This is consistent with our own observations made in a patient with heterozygous pathogenic gene variant in CD48 suffering from recurrent hyperinflammation (25). We have shown that reduced CD48 expression leads to an increased resistance of human APCs to killing. This provides evidence that immunostimulatory APCs contribute to the pathogenesis of HLH, also in humans. A hallmark of HLH is macrophage activation. On one hand excessive cytokine production by CTLs leads to macrophage activation in HLH. On the other hand, a defect in cytokine control, as in NLRC mutations and ZNFX1 deficiency can, independently of CTLs, lead to macrophage activation in an HLH context. Disease mechanisms linking deficient cytotoxicity and deficient cytokine control to HLH spectrum diseases are summarized in **Figure 2**.

### 5.1 Dysregulation of CD48-triggered SLAMF-dependent cytotoxicity in HLH

**Figure 3:**
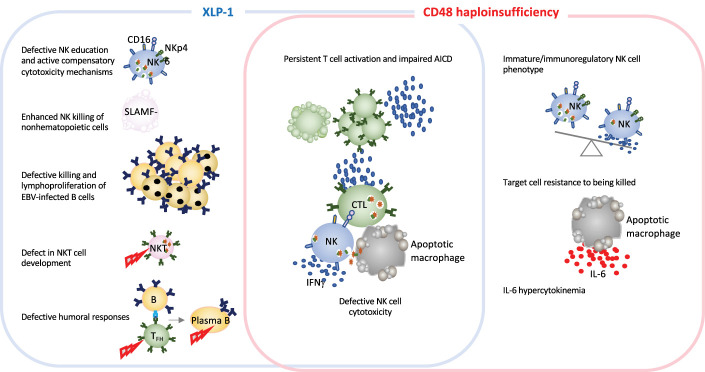
Schematic representation of immune defects in two related inborn errors of cytotoxicity: XLP-1 (SAP deficiency) and CD48 haploinsufficiency. In humans and animal models, XLP-1 and CD48 haploinsufficiency are related because SAP is an adapter molecule for the high-affinity CD48 receptor. However, the two diseases also present differences. XLP-1 disorder in humans and/or SAP deficiency in mice (in the blue box) cause NK cell abnormalities, such as the defective killing of EBV-infected target cells, subsequent EBV-infected B cell lymphoproliferation and NK education defects. These affects are partially countered by compensatory NK killing mechanism. Profound NKT cell developmental defects and defective humoral responses are also hallmarks of XLP-1. SAP deficiency and CD48 haploinsufficiency (in the red box) trigger some of the same generic dysregulations, such as defective NK cell cytotoxicity and impaired activation-induced cell death. Furthermore, CD48 haploinsufficiency impairs the maturation of NK cells. Given that CD48 is a ligand of the cytotoxicity triggering receptor 2B4, CD48 haploinsufficiency enables the target cell to better resist granule-mediated cytotoxicity. Moreover, the hypercytokinemia in this disease is characterized by a high serum IL-6 level, as observed in a CD48 haploinsufficient patient and the CD48^-/-^ mouse model (11).

As in the case for other IEI, monogenic HLH diseases are excellent models for improving our knowledge of how the immune system works. In-depth studies of immune dysregulation in patients with HLH and animals models of conventional HLH have provided invaluable information on the important role of granule-mediated cytotoxicity in eliminating virus-infected cells and in terminating immune responses. The newly identified CD48 haploinsufficiency and XLP-1 are clinically distinct but immunologically related disorders. Both CD48 and SAP mutations affect the 2B4-triggered cytotoxicity pathway, by either diminishing cell-surface expression of the 2B4 ligand or affecting the stability or binding capacity of a signaling adapter molecule (14, 79). SAP and CD48 pathogenic gene variants cause partially overlapping features, such as cytotoxicity defects and hyperinflammation. However, the disorders differ with regard to clinical signs, the extent of cytotoxicity impairment, and the immune compensatory mechanisms (**Figure 3** and **Table 1**). While in XLP-1 all SAP-dependent pathways are impaired, in CD48 haploinsufficiency only CD48-triggered 2B4-dependent SAP signaling is affected. This is probably the underlying mechanism setting the differences between these two disorders.

The viral triggers associated with HLH and hyperinflammatory syndromes are mainly DNA herpesviruses (such as EBV and human cytomegalovirus (CMV)) and more rarely herpes simplex virus, human herpesvirus 6 and 8, and varicella zoster virus (9). Hyperinflammatory episodes in CD48 haploinsufficiency are not currently known to be associated with greater vulnerability to a specific pathogen (14). CD48 haploinsufficiency appears to be less aggressive clinically and immunologically than XLP-1. This novel disorder is characterized by normal perforin-dependent T cell cytotoxicity and an NK cell degranulation towards K562 cells within reference values but lower cytotoxicity towards autologous EBV-immortalized lymphoblastoid cells. Phenotypic and functional analyses of the NK cells of patient with CD48 haploinsufficiency have nevertheless evidenced an impact of CD48 on NK cell maturation, since the patient presented a high number of immunoregulatory-immature CD56^bright^ CD94^high^ NK cells, high levels of IFNγ production by NK cells upon IL-12 stimulation, and a low number of the more differentiated, highly cytotoxic CD56^dim^ CD94^low^ cells. Interestingly, a similarly immature NK profile has been observed in patients with an IEI affecting the IL-2/CD25 pathway (39). It is noteworthy that HLH-episodes in CD48 haploinsufficiency were not associated with high serum sCD25 levels as opposed to the high levels observed in FHL (14).

A remarkable novelty that CD48 haploinsufficiency provided to HLH knowledge is the importance of the target cell in perforin-dependent cytotoxicity. Of note, CD48 is expressed on both the cytotoxic cell and the target cell. In CD48 haploinsufficiency, the target cells themselves might have an active role in the pathogenesis of HLH because CD48 expression might be a “kill me” signal as a ligand for 2B4 receptors on the killer cell promoting cytotoxicity. A similar mechanism has been described for controlling activated CD155-expressing T cells upon DNAM-1 interaction on NK cells, in the context of immune regulation in autoimmunity (156). Indeed, cell-surface CD48 expression in target cells determines the degree of susceptibility to NK cell killing in hyperinflammation, as shown by experiments on cell lines transfected with a plasmid encoding wildtype and mutated CD48 or experiments in which the target cell is pre-coated with blocking antibodies (14). The interactive feedback loop between target cells and cytotoxic lymphocytes is crucial for the elimination of APCs and a return to immune homeostasis (154).

CD48 signaling defects affect also T lymphocytes. In a CD48 knock-out model, low CD4^+^ and CD8^+^ T cell proliferation has been observed (157). However, it must be born in mind that CD48 has an additional low affinity receptor (CD2) which, in humans, has a high affinity ligand (CD58); this is not the case in mice. In patients, it has not yet been established whether CD48-associated alterations are mediated by low-affinity CD2 receptor signaling. AICD is impaired in CD48 haploinsufficiency, as in SAP mutations (158, 159). CD48 protects against restimulation-induced cell death by maintaining basal autophagy and inhibiting p53 signaling in a SAP-independent manner (160). Finally and concerning the humoral branch of the adaptive immune system, CD48 haploinsufficiency causes slight alterations in IgG subtypes (such as low IgG2 levels) but not profound hypogammaglobulinemia (14).

### 5.2 Dysregulated cytokine control in novel HLH spectrum disorders

HLH is characterized by hypercytokinemia and systemic inflammation. IFNγ is a key pathogenic cytokine in both pHLH and MAS (161) and is produced by overactivated CTLs (67, 68). In pHLH, a mouse model with genetically depleted IFNγ demonstrates that this cytokine is responsible for the hematologic features of the disease, such as anemia (162). Indeed, the monoclonal anti-IFNγ antibody emapalumab is the first cytokine-targeted therapy approved for pHLH patients that have a refractory or relapsing disease or do not tolerate first-line therapies (63, 163). This contrasts with low IFNγ responses to EBV-infected B cells in patients with XLP-1 (164). Patients with XLP-2 present elevated levels of cytokines such as IFNγ, IL-6, TNFα and IL-18 (106).

The cytokine IL-33 is released by stressed and necrotic cells. Thus, it is considered an “alarmin” that activates immune responses upon injury. IL-33 binds its receptor ST2 resulting in a MyD88-dependent signaling. In the context of HLH, experimental models blocking ST2 have demonstrated that IL-33 enhances the IFNγ-driven pathology in HLH (165).

IL-6 is another proinflammatory cytokine associated with HLH hyperinflammation. In CD48 haploinsufficiency, PBMCs upregulate the linker for activation of T cells-regulated cytokine and phospholipase Cγ transcript upon activation (14). This disorder is associated with IL-6 hypercytokinemia, both in the patient with CD48 deficiency and in the CD48 knock-out mouse model upon LCMV infection. In HLH, defective killer cell-target cell disengagement promotes persistent activation of the killer cells and the production of proinflammatory cytokines that induce IL-6 secretion by macrophages. Interestingly, IL-6 hypercytokinemia and cytotoxicity defects were observed during the cytokine storms in severe COVID-19 (166) and MAS (167) – two hyperinflammatory disorders with overlapping features. Monoclonal antibodies against the IL-6 receptor, IFNγ and Janus kinases 1 and 2 (which act downstream of the cytokines) are being tested as an immunomodulatory treatment for HLH (81, 163, 168, 169). In MAS, lack of cytotoxicity-induced apoptosis in target cells favors the lytic death of the latter; this releases alarmins that activate inflammasomes and leads to a hyperinflammatory situation (170).

**Figure 4:**
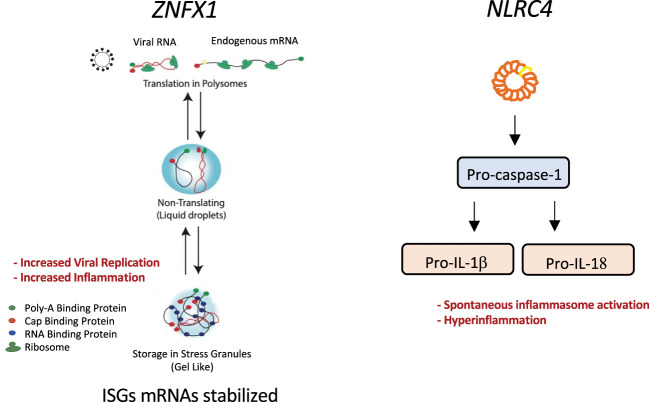
Graphical representation of the molecular pathways affected by two inborn errors of cytokine control leading to HLH: *ZNFX1* and *NLCR4* pathogenic gene variants. ZNFX1 deficiency (left panel) causes susceptibility to viral infections. At later stages of infection, increased expression of interferon-stimulated genes is driven by greater mRNA stability; this prevents a return to homeostasis and leads to hyperinflammation and multiple organ failure. ZNFX1 localizes to stress granules. We hypothesize that ZNFX1’s helicase function is needed to remove mRNAs stored in stress granules formed upon viral infection. In the absence of ZNFX1, the more stable mRNA is translated and not degraded, preventing a return to homeostasis and leading to a hyperinflammatory syndrome. *NLRC4* mutations can cause intrinsic macrophage activation (right panel). A mutation in the nucleotide-binding domain of the NLRC4 sensor activates inflammasomes and leads to pro-caspase-1 cleavage. Subsequent caspase-1 dependent activation of pro-IL-1b and pro-IL-18 results in macrophage activation and hyperinflammation.

Mutations in genes involved in the regulation of cytokine transcription have been recently linked to HLH (**Figure 4**). NLRC4 is an intracellular pattern recognition receptor that activates inflammasomes. NLRC4-triggered inflammasomes induce the activation of caspase 1 and the production of proinflammatory cytokines IL-1β and IL-18 (171). *NLRC4* pathogenic gene variants have been linked to spontaneous inflammasome activation and IL-1β and IL-18 hypercytokinemia (16). An imbalance between IL-18 and its natural antagonist (IL-18 binding protein) might be related to the development of MAS (172). In contrast, IL-10 appears to have a protective role (112). CXCL9 is an inflammatory chemokine induced by IFNγ recently linked to MAS as disease biomarker (173, 174). In addition to the cytokine effect per se, novel hyperinflammatory disorders with HLH disorders highlight the importance of post-transcriptional RNA regulators of cytokine transcripts to control hyperinflammation. For instance, genetic mutations responsible for a deficiency in roquin-1 (a regulator of RNA transcripts involved in immune responses) can also cause a dysregulation in cytokine synthesis and lead to hyperinflammation as described above for ZNFX1 (18).

## 6 Concluding remarks and open questions

A better understanding of the genetic disease mechanisms underlying HLH is essential for more accurate, more personalized diagnoses. Pathway-related pathogenic gene variants associated with HLH (such as inborn errors of cytotoxicity or cytokine control) can manifest themselves to different extents with regard to aggressiveness, clinical signs, and immune cell defects.

CTLs and NK cells are crucial cytotoxic lymphocytes for controlling overactivated immune responses, as has been demonstrated for HLH. NK cells have been extensively studied in HLH and are widely used to screen for cytotoxicity and degranulation defects in HLH (175). In the future, research should also focus on the roles of CD8^+^ CTLs and unconventional cytotoxic T cells (such as polyclonal cytotoxic CD4^+^ T terminally differentiated cells, which are enriched in pHLH) (176, 177).

Two novel HLH disease mechanisms have been revealed by the identification and characterization of additional monogenic immune disorders with HLH. The target cell’s active role in susceptibility to killing (by regulating the expression of cytotoxicity receptor ligands) is a novel HLH-associated dysregulation seen in CD48 haploinsufficiency. CD48 is also a candidate molecular target for controlling not only HLH but also autoimmunity (156), and cancer (178). The cell-intrinsic production of cytokines caused by inborn errors of cytokine control might also improve our understanding of systemic hyperinflammation in other fields, such as autoinflammatory diseases or severe COVID-19.

The enormous power of whole-exome and whole-genome sequencing will certainly help to identify links between novel genetic mutations and HLH in the future. However, in order to confirm novel genetic associations and characterize their phenotypical consequences, it will still be necessary to continue the development of functional immunological assays and animal models. For instance, novel autoinflammatory syndromes with HLH due to IEI evidence the need to better understand what to what extend HLH and autoinflammation overlap, in order to provide disease-specific treatments. Hypercytokinemia contributes to link this two disorders, with a predominant role for IL-18 and IL-1 in autoinflammation that enhance the production of IFNγ, key effector molecule in HLH immunopathology. Translational studies from patients with autoinflammation and/or HLH responsive to cytokine oriented therapies will help dissecting the role for IL-18, IL-1, IFNγ and others (IL-6) in different hyperinflammatory disorders. In addition to IFNγ hypercytokinemia, viral triggers, poor viral control -sometimes associated with IEI- and defects in cytotoxic lymphocytes are other key factors more specifically contributing to HLH development. Larger, collaborative studies of patients with the novel genetic disorders with HLH will also be essential for defining typical and atypical disease manifestations and developing personalized therapies.

## Author contributions

RP wrote the manuscript and prepared figures. MF collected and visualized clinical data and figures. SV contributed to manuscript writing and prepared figures. JPS wrote the manuscript and prepared figures. All the authors reviewed and edited the manuscript. All authors contributed to the article and approved the submitted version.
